# In Operando Characterization and Control over Intermittent Light Emission from Molecular Tunnel Junctions via Molecular Backbone Rigidity

**DOI:** 10.1002/advs.201900390

**Published:** 2019-08-22

**Authors:** Tao Wang, Wei Du, Nikodem Tomczak, Lejia Wang, Christian A. Nijhuis

**Affiliations:** ^1^ Department of Chemistry National University of Singapore 3 Science Drive 3 117543 Singapore Singapore; ^2^ Institute of Materials Research and Engineering A*STAR (Agency for Science, Technology and Research) 2 Fusionopolis Way, Innovis 138634 Singapore Singapore; ^3^ Centre for Advanced 2D Materials and Graphene Research Centre National University of Singapore 6 Science Drive 2 117546 Singapore Singapore; ^4^ NUSNNI Nanocore National University of Singapore 117411 Singapore Singapore

**Keywords:** intermittent light emission, molecular electronics, molecular tunneling junctions, noise characterization, plasmon excitation

## Abstract

In principle, excitation of surface plasmons by molecular tunnel junctions can be controlled at the molecular level. Stable electrical excitation sources of surface plasmons are therefore desirable. Herein, molecular junctions are reported where tunneling charge carriers excite surface plasmons in the gold bottom electrodes via inelastic tunneling and it is shown that the intermittent light emission (blinking) originates from conformational dynamics of the molecules. The blinking rates, in turn, are controlled by changing the rigidity of the molecular backbone. Power spectral density analysis shows that molecular junctions with flexible aliphatic molecules blink, while junctions with rigid aromatic molecules do not.

## Graphical abstract

**In molecular tunnel junctions, plasmons are excited via inelastic tunneling where the charge carriers tunnel through molecular bonds.** These junctions blink due to molecular vibrations and conformational changes. This article shows that the light emission is unstable for junctions with flexible aliphatic backbones while junctions with rigid molecular backbones do not blink paving the way for stable molecular plasmon sources.

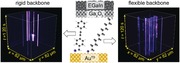

## 1 Introduction

Surface plasmon polaritons (SPPs) can carry optical information at petahertz frequencies along sub‐diffractive metallic waveguides, which enables new designs for integrated optoelectronic devices.^1^, ^2^, ^3^ Although the optical properties of the plasmonic structures, such as metallic particles, meta‐surfaces, and waveguides, have been extensively studied,^4^, ^5^, ^6^, ^7^ there is still a lack of robust on‐chip plasmon sources that convert electrical signals to plasmonic signals.^8^, ^9^, ^10^, ^11^, ^12^, ^13^, ^14^, ^15^, ^16^, ^17^ To overcome this challenge, traditional light sources, such as light‐emitting diodes (LEDs), can be miniaturized and integrated with plasmonic waveguides.^8^, ^9^, ^10^, ^11^, ^12^ Another approach is to use tunnel junctions as on‐chip plasmon sources, that directly excite plasmons by electrons that tunnel inelastically.^13^, ^14^, ^15^, ^16^, ^17^ In the former approach, plasmons are excited in two steps involving a relatively slow electron–hole recombination process (>ps),^18^ while in the latter approach plasmons are excited in a single step at much faster quantum tunneling timescales (≈fs).^14^, ^15^, ^17^, ^19^

Recently, we have demonstrated on‐chip electrically driven plasmon sources using self‐assembled monolayer (SAM)‐based tunnel junctions (STJs) and showed the control over the plasmonic properties (such as intensity, polarization, and frequency) by the chemistry of the molecules in the STJs.^20^ Under applied bias, the STJs excite surface plasmons from a discrete number of diffraction‐limited spots, which blink between “on” (bright) and “off” (dark) states with on‐ (*t*_on_) and off‐ (*t*_off_) times ranging from microseconds to minutes. We characterized the on‐time (*P*(*t*_on_)) and off‐time (*P*(*t*_off_)) probability densities of the intermittent light emission (blinking) in the STJs and found that the *P*(*t*_on_) and *P*(*t*_off_) probability densities follow a power‐law^20^ dependence similar to other point‐like sources, such as single chromophores or quantum dots.^21^ Here, we reveal the underlying mechanism that causes blinking in STJs and how it relates to the conformational changes of molecules in the junction. We use this insight to control the intermittency of the light emission by changing the molecular backbone rigidity (defined as the amount of energy required to rotate the molecular backbone around the C‐C bond, see Supporting Information and also below). These findings are important for the field of molecular electronics as it is explicitly demonstrated here that the dynamic aspects of the molecule in‐between the electrode interfaces must be taken into account in device engineering.^22^, ^23^, ^24^, ^25^, ^26^, ^27^, ^28^

In molecular electronics, a challenge is to rationalize the large spread of eight orders of magnitude in the measured values of *J* (the current density) for the same molecule incorporated in different types of junctions.^28^, ^29^, ^30^, ^31^, ^32^, ^33^, ^34^, ^35^, ^36^, ^37^ Although it has been proposed that this discrepancy can be (largely) explained by assuming that the effective electrical contact area between the SAM and the top electrode is orders of magnitude smaller than the geometrical contact area, reliable methods to measure the effective electrical contact area are not available.^28^, ^30^, ^38^ It is well‐known that the effective electrical contact area between two solid surfaces can be several orders of magnitude smaller than the geometrical contact area due to the surface roughness of the electrodes, in molecular electronics, however, it is common practice to assume that all molecules participate in charge transport and that the top‐electrode always makes good contact to the molecules regardless of their chemical nature. In addition, the dynamics of molecule–electrode contacts are usually ignored and assumed to be static, while molecules have of course conformational degrees of freedom depending on their chemical structure.

We believe that the intermittent light emission of molecular electronic plasmon (MEP) sources can be explained by the fluctuating barrier model associated with the conformational changes of the molecules in response to inelastic tunneling where charge carriers interact with the vibrational modes of the molecule resulting in conformational changes of the molecules.^39^ As a result of these molecular conformational changes, the effective tunneling barrier width and height fluctuate resulting in current fluctuations. The dynamics of the molecular conformational changes has been well‐studied in molecular tunnel junctions^40^, ^41^, ^42^, ^43^, ^44^, ^45^, ^46^, ^47^, ^48^ and followed in real‐time by recording the change of tunneling current as a function of time.^40^, ^42^, ^43^, ^44^, ^45^, ^46^, ^47^, ^48^ Zandvliet et al. demonstrated convincingly that inelastic excitation‐induced conformational changes can be used to trap single molecules between a scanning tunneling microscope (STM) tip and a surface.^49^, ^50^

Under continuous excitation, point sources, such as quantum dots and single molecules, emit light irregularly, and often switch between “on” and “off” states with the times *t*_on_ and *t*_off_ that characterize the length of each state ranging from microseconds to minutes.^21^, ^51^, ^52^, ^53^, ^54^ The *P*(*t*_on_) and *P*(*t*_off_) follow an inverse power law, $P(t_{\text{on}})\propto t^{-m_{\text{on}}}$ and $P(t_{\text{off}})\propto t^{-m_{\text{off}}}$, where *m*_on_ and *m*_off_ range from 1 to 2.^21^, ^51^, ^52^, ^53^, ^54^ This power‐law distribution holds over many orders of magnitude of *P* and *t*.^52^ Another important feature of blinking is the power‐law dependency of the power spectral density *S*(*f*).^21^, ^55^, ^56^, ^57^ In contrast to the *P*(*t*) analysis, *S*(*f*) is in the frequency domain and is the Fourier transform of the autocorrelation of the emission intensity time trace *I*(*t*).^55^, ^56^, ^57^ Similar to *P*(*t*), the distributions of *S*(*f*) follow a power law in the form of *S*(*f*  ) ∝ *f* ^−α^ where α is between 1 and 2.^55^, ^56^, ^57^ The *S*(*f*) behavior is often modeled with Equation (1)

(1) $$S(f)\;=\;\frac{A}{f}+\frac{B}{1+{\left(f\text{/}f_{0}\right)}^{2}}+C$$

where *A* and *B* are the amplitudes of the 1/*f* (also called flicker noise) and the 1/*f* ^2^ components, *f*_0_ is the characteristic frequency of the 1/*f* ^2^ component and can be characterized by the inflection point in *S*(*f*), and the constant *C* does not depend on frequency and is an offset representing the white noise floor.^29^ Equation (1) is widely used to evaluate the fluctuations in time traces, and has been used to understand the intermittent light emission of point sources.^55^, ^56^, ^57^

In this work, we experimentally demonstrate the control of the intermittent light emission of the MEP sources by the rigidity of the molecular backbone. With stiff aromatic molecular backbones, the blinking is almost eliminated, while the opposite is true for flexible aliphatic molecular backbones over the range of frequencies (10^−3^–10^0^ Hz) studied here. These observations suggest that conformational changes of the molecules play a major role in the blinking of MEP sources. This work also highlights that the electrical contact between the electrode and the barrier molecules is highly heterogenous and varies from junction to junction depending on the chemical nature of the molecules. In addition, the electrical contact points along which tunneling occurs change within the junctions resulting in time‐dependent changes in the junction properties.

## 2 Results and Discussion

**Figure 1:**
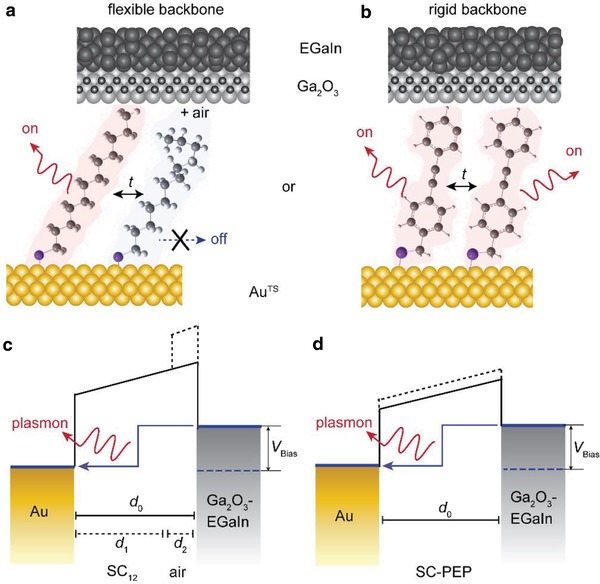
Schematic illustrations of two types of STJs based on a) SC_12_ and b) SC‐PEP molecules, and c,d) the corresponding energy level diagrams. Panel (a) indicates how due to a conformational change, a Gauche defect forms disconnecting the SC_12_ molecule from the top electrode and panel (c) shows the corresponding change in the tunneling barrier shape. Panel (d) indicates the energy level diagram for the junctions with SC‐PEP molecules; current fluctuations are still possible due to phenyl ring rotations (see the text for details). The blue arrows in (c,d) indicate inelastic electron tunneling from the Ga_2_O_3_‐EGaIn electrode to the Au substrate which results in the excitation of plasmons.

**Figure** **1** shows the two types of STJs with two different types of molecular backbones. We used STJs with SAMs of dodecane‐1‐thiolate (in short SC_12_, Figure 1a) and (4‐(phenylethynyl)phenyl)methanethiolate (in short SC‐PEP, Figure 1b). Because of the sp^2^ character, the SC‐PEP molecules have a more rigid molecular backbone than the sp^3^ aliphatic SC_12_ molecules.^22^, ^40^ Following the same methods (see the Supporting Information) as described by Du et al.,^20^ we prepared the two types of STJs using semitransparent 50 nm gold bottom electrodes obtained by template‐stripping onto which we formed the SAMs. The top electrodes were formed using EGaIn (a eutectic gallium‐indium alloy with 0.7 nm native Ga_2_O_3_), which is well‐established and generates high‐quality molecular junctions in high yields of working devices.^30^, ^31^, ^38^, ^58^, ^59^ EGaIn is a non‐Newtonian liquid metal that flows when shear pressure is applied.^60^ When brought in contact with the SAM, EGaIn flows and deforms for an instant, before solidifying again. This feature of EGaIn ensures that a soft top‐contact is generated and that the SAMs retain their supramolecular structure, essential to obtain a good tunneling barrier. In our experiments, the EGaIn was confined in a through‐hole in a polydimethylsiloxane microfluidic device and the top‐electrode had a geometrical contact area of 1000 µm^2^.

Figure 1 also shows schematically the difference in the conformational degree of freedom between SC_12_, which, e.g., can have gauche conformations which are not possible for SC‐PEP. Consequently, an SC_12_ molecule can be in contact with both electrodes when in an all‐trans conformation but the contact with the top‐electrode will be lost when a Gauche defect is present. In the former case, the tunneling barrier width (*d*_0_) and height and the corresponding tunneling decay coefficient (β_1_) are defined by the molecule (solid line in Figure 1c). In the latter case, a gap between the molecule and the top‐electrode is present and, consequently, besides the molecular component (defined by *d*_1_ and β_1_) a second component is presently defined by the air gap *d*_2_ and associated tunneling decay coefficient β_2_ (dashed line in Figure 1c). Since through‐molecular bond tunneling is more efficient than tunneling through air, the local tunneling current decreases significantly along the molecule when an air gap forms. Hence, the local current increases elsewhere inside the junction until the molecule changes its conformation again since the total current across the junction remains the same with variations within noise levels (see previous works for *J*(*t*) measurements^20^, ^31^, ^38^). Therefore, when the current across the molecule is low, the plasmon excitation rate (and thus the photon emission rate) is low, and vice versa. In other words, this conformational change (and other types of conformational changes) results in a fluctuating tunneling barrier and consequently in intermittent light emission. Although SC‐PEP molecules are “stiff,” conformational changes can still lead to current fluctuations due to, for instance, rotation of the phenyl rings (Figure 1d). It is well known that when the two phenyl groups are out of plane (large dihedral angles), the conductance along the molecule can be up to ten times smaller than when the phenyl groups are in plane (small dihedral angles).^61^, ^62^ In the SAM, however, these rotations are hampered.^63^, ^64^

**Figure 2:**
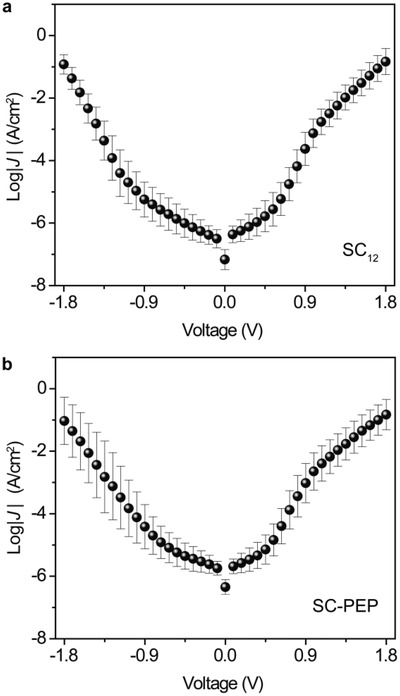
*J*(*V*) curves of STJs with a) SC_12_ and b) SC‐PEP SAMs in the bias range of ±1.8 V. The *J*(*V*) values are averaged over eight junctions, and the error bars are the corresponding log standard deviations determined over 150 *J*(*V*) traces.

**Figure 3:**
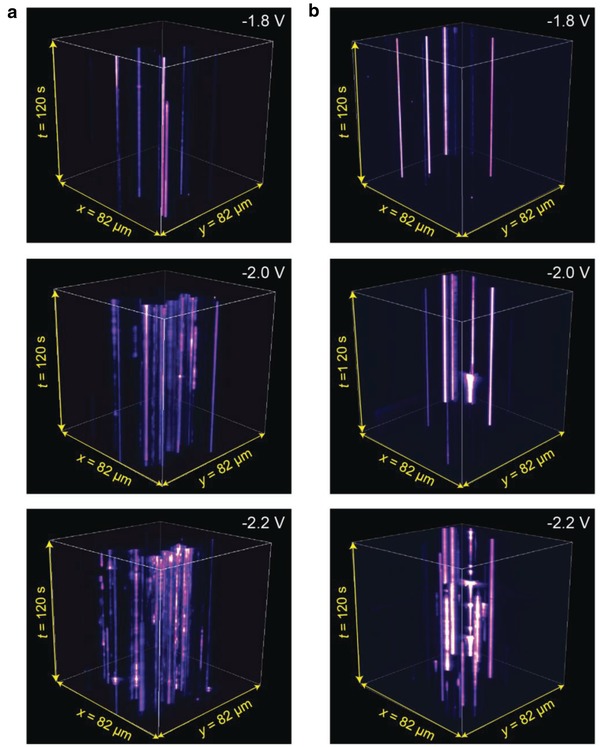
Intermittent light emission from the two STJs. Real plane images (*x*–*y* plane) placed on top of each other as a function of time *t* (*z*‐axis) for a) SC_12_ and b) SC‐PEP junctions. The images were recorded at intervals of 1 s at a bias of −1.8, −2.0, and −2.2 V, with 300 EM Gain. The optical spatial resolution of the set‐up is 160 nm per pixel.

The *J*(*V*) characteristics of the two types of STJs were recorded using the Keithley 6430 source meter and a home‐made Labview program, with electrical bias applied between −1.8 and +1.8 V in steps of 90 mV. **Figure** **2** shows the log‐average *J*(*V*) curves (averaged over 150 traces) of the two types of STJs. We note that the two SAMs have a similar molecular length (1.8 nm for SC_12_ and 1.6 nm for SC‐PEP), thus junctions with SC‐PEP SAMs with the aromatic molecular backbone, in theory, should have higher *J* values than junctions with SC_12_ SAMs because aromatic molecules have smaller tunneling decay coefficients (0.2–0.4 Å^−1^) than aliphatic molecules (0.8 Å^−1^),^32^, ^33^, ^34^, ^35^, ^36^ see Equation (2) below. This discrepancy can be explained by the difference in the effective electrical contact area which is directly visualized in the light emission images from the two types of STJs shown in **Figure** **3** (and see below for more details).

**Figure 4:**
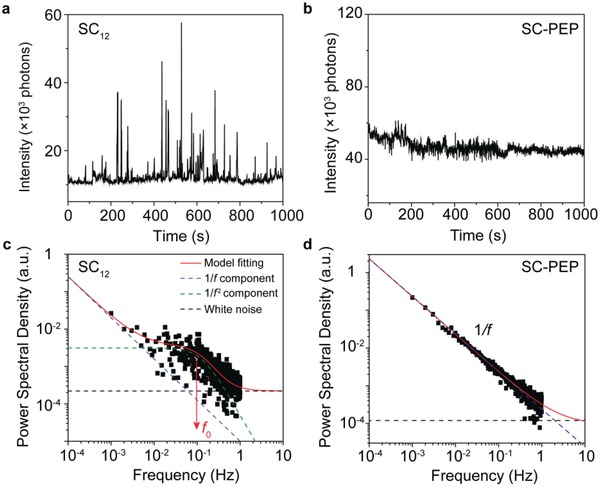
Representative emission intensity time traces of the STJs with a) SC_12_ and b) SC‐PEP SAMs recorded at an applied bias of −2.0 V at 0.5 s time intervals. The corresponding *S*(*f*) analysis of the two emission intensity time traces is plotted in (c) and (d).

**Figure 5:**
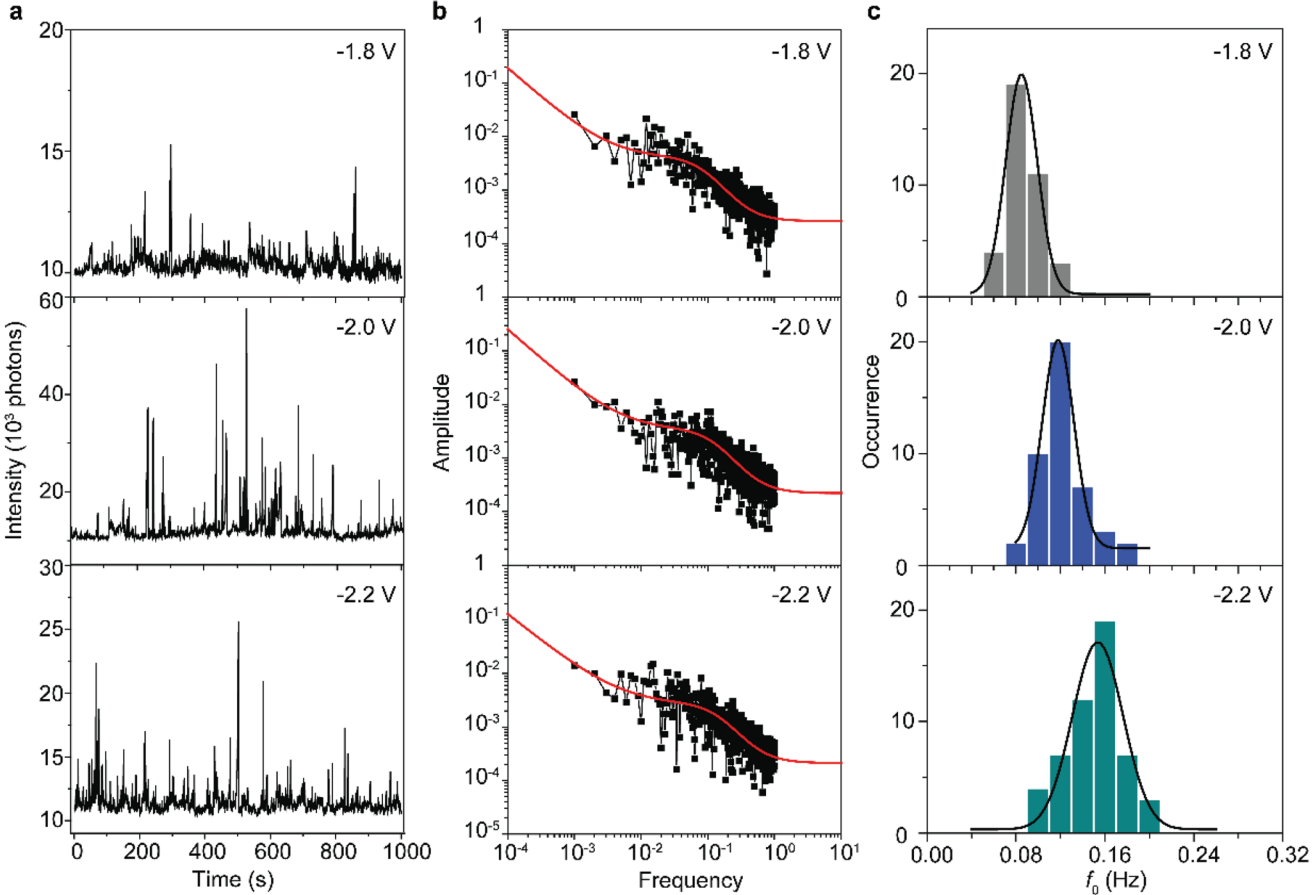
The bias dependency of the intermittent light emission of junctions with SC_12_ SAMs. a) Time traces of the light emission intensity at different biases. b) *S*(*f*) analysis of the time traces in (a). c) The corresponding histograms of the *f*_0_ with Gaussian fits to these histograms. The histograms are obtained from the measured emitting spots of four samples using videos consisting of 2000 frames.

To record the light emission from the STJs in real‐time, we used a wide‐field inverted optical microscope equipped with an electron‐multiplying charge coupled device and a 100 × oil immersion objective with the numerical aperture NA = 1.49 (see the Supporting Information). Figure 3 shows real plane emission images of the two STJs recorded over a period of time of 120 s (1 s per image) at −1.8, −2.0, and −2.2 V bias (see also Figures S1–S3, Supporting Information for additional light emission images and spectra). By comparing Figure 3a,b, we make the following observations: i) the emission spots in STJs with SC‐PEP SAMs blink less frequently than STJs with SC_12_ SAM (see also **Figure** **4**a,b and Supporting Movies, Supporting Information), ii) the blinking rate increases with increasing bias for both types of STJs (see also **Figure** **5** and Figure S4, Supporting Information), and STJs with SC‐PEP SAMs have a smaller number of emission spots than STJs with SC_12_ SAMs. In the following sections, we discuss this in detail. We note that the area with no emission cannot be caused by “quenched electroluminescence” of the molecules as the highest occupied molecular orbital–lowest unoccupied molecular orbital gap of the molecules is much larger (4.5 eV for SC‐PEP^65^ and 8–9 eV for SC_12_ 20, 35) than the applied voltage bias. The emission spots directly represent the effective electrical contact points of the top‐electrode with the SAM where the current flows and plasmons are excited via inelastic tunneling. For the areas without light emission, the top‐electrode is not in contact with the SAM and, hence, no current flows in that area. The main reason for the low effective electrical contact areas in EGaIn junctions is that due to the non‐Newtonian properties of the GaO*_x_*/EGaIn, the surface of GaO*_x_*/EGaIn contains wrinkles which reduce the contact area on the macroscopic scales.^66^ Due to the polycrystalline nature of the GaO*_x_* layer, microscopic roughness is also present which reduces the effective contact area further explaining the low effective contact areas we report here.^67^ It should be noted that intermittent light emission as a result of charge trapping involving the GaO*_x_* is not important as no hysteresis is observed in the *J*(*V*) curves and clear molecular effects are resolved as in our experiments the top‐electrodes were fabricated always in exactly the same manner. We have shown elsewhere that the 0.7 nm thin GaO*_x_* layer shows metallic behavior and only has a minor contribution to the contact resistance.^31^, ^38^

To quantify the blinking dynamics, we performed *S*(*f*) analysis of the intermittent light emission from individual emission spots for both types of STJs (see Figure S5, Supporting Information). Figure 4 shows representative time traces of the light emission intensity recorded over 1000 s at an applied bias of −2.0 V along with the *S*(*f*) analysis. The *I*(*t*) of the STJ with SC_12_ SAMs (Figure 4a) shows clear jumps (>10k counts) between the on and off states. However, the emission of the STJ with SC‐PEP SAMs (Figure 4b) remains in the “on” state and displays small variations in intensity (<5k counts). Within the experimental frequency range (10^−3^–1 Hz), the *S*(*f*) of the SC_12_ junction (Figure 4c) decreases with increasing *f* and has a maximum value of around 0.1 Hz. The solid red line is a fit to Equation (1) with three components: the 1/*f* component at the low frequencies, the 1/*f* ^2^ component with *f*_0_ = 0.1 Hz, and the white noise component (at the level of 2 × 10^−4^ a.u.). In contrast, the *S*(*f*) of the SC‐PEP junction (Figure 4d) decreases as a function of *f* and only has the 1/*f* and the white noise components (at the level of 1 × 10^−4^ a.u.). Here, the 1/*f* component results from intensity fluctuations that occur randomly in the on state and do not have a measurable repetition period. While in the case of SC_12_ junctions, the 1/*f* component is mainly due to the random intensity fluctuations of the off states. The extra 1/*f* ^2^ component with the characteristic frequency *f*_0_ = 0.1 Hz corresponds to the pronounced intensity fluctuations (the blinking) between the on and off states with a repetition period of around 10 s. It is important to note that although the time scale for the molecular conformational change can be very fast, the conformational changes in SAM are very slow due to steric hindrance between neighboring molecules and favorable packing energies. Previous STM experiments^50^, ^68^, ^69^ show that the alkanethiol and oligo(*p*‐phenyleneethynylene) molecules in SAMs keep their conformations stable over prolonged periods of time up to hours.

It has been reported before that the rate of conformational changes induced by inelastic tunneling increases with increasing applied bias.^42^, ^43^, ^44^, ^45^, ^46^, ^47^, ^48^, ^49^, ^50^ Therefore, we studied the bias dependency of the blinking behavior by determining *S*(*f*) of the SC_12_ junctions at −1.8, −2.0, and −2.2 V. Figure 5 shows representative time traces of the light emission intensity along with the *S*(*f*) analysis. For each applied bias, we analyzed 40–50 individual blinking spots to determine *f*_0_ and Figure 5c shows the histograms of *f*_0_ with a Gaussian fit (because the blinking of the emission spots is not correlated, but random) to these histograms to determine the Gaussian mean value of *f*_0_. The value of *f*_0_ increases from 0.09 Hz at −1.8 V to 0.15 Hz at −2.2 V showing that the blinking rate increases with bias. These observations agree with inelastic tunneling: with increasing bias both the tunneling electron energy and the tunneling electron rate increase, thus the possibility for the conformational change increases, which explains the near doubling of *f*_0_. For SC‐PEP junctions, we also observed an increase in the blinking rate with increasing bias, however, the 1/*f* component dominates in the measured bias range (see Figure S4, Supporting Information).

**Figure 6:**
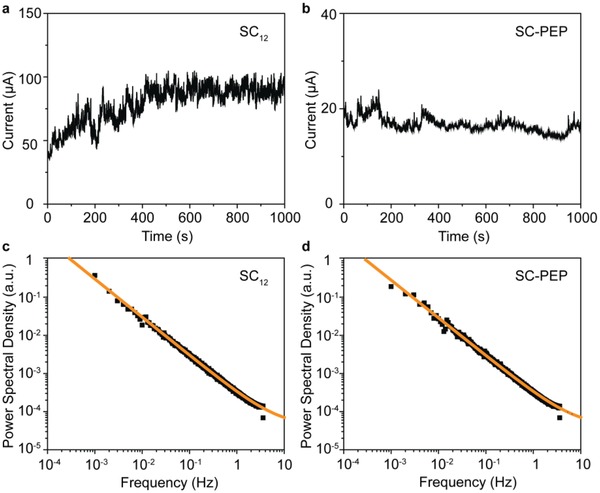
The current‐time traces of an STJ with a )SC_12_ SAMs and b) SC‐PEP SAM at an applied bias of −2.0 V and c,d) the corresponding *S*(*f*) analysis.

To investigate how the tunneling current influences the blinking of the light emission in STJs, we also performed the *S*(*f*) analysis of the time traces of the tunneling current. **Figure** **6** shows the tunnel current versus time traces of the STJs with SC_12_ SAMs and SC‐PEP SAMs with the current sampling time 0.13 s (3.8 times faster than the optical case, 0.5 s). For both types of STJs, the tunneling current fluctuations are smaller than 20% and only show the 1/*f* component. This cannot account for the 1/*f* ^2^ blinking behavior of the SC_12_ junction. We note that the recorded tunneling current represents the current flowing across all spots in the entire STJ. As shown in Figure 3 and the Supporting Videos (Supporting Information), the blinking of the emission spots is not correlated. Consequently, the local current fluctuations are also not correlated and therefore current fluctuations at local spots cancel each other within the noise level of the measured total currents (see Figure S6, Supporting Information). Thus, the advantage of the optical characterization becomes obvious as it allows for independent analysis of each individual light‐emitting spot providing local information on how the current flows across an individual spot at any given time. We also note that, in tunnel current time trace measurements, the term *C* represents the thermal noise of the tunnel junctions. For both types of junctions, the current is at the same level (within a factor 2–4) and the term *C* of 5 × 10^−5^ is also very similar (Figure 6c,d).

We estimated the size of the air gap required to induce a significant change of the local current for the SC_12_ molecule (Figure 1c) as follows. The value of *J* can be described by the general tunneling equation (Equation (2))

(2) $$J\;=\;J_{0}e^{-\beta_{1}d_{0}}$$

The tunnel barrier can be described as a double barrier junction by Equation (3) with a gap of air

(3) $$J^{\prime }\;=\;J_{0}e^{-\beta_{1}d_{1}}e^{-\beta_{2}d_{2}}\;=\;J_{0}e^{-\beta_{1}(d_{0}-d_{2})}e^{-\beta_{2}d_{2}}$$

where β_2_ is the tunneling decay coefficient of air (since our experiments are conducted in ambient conditions). Since the value of β_2_ of air is 2.9 Å^−1^ and β_1_ is 0.8 Å^−1^ for aliphatic SAMs,^30^, ^34^, ^35^, ^36^, ^38^ a value of *d*_2_ of 1.1 Å (which is roughly the length of one CH_2_ unit) results in a factor of 10 decrease in the value of *J*. Such a small gap can be easily obtained by bending of the molecule and (partial) rotation of molecular segments around C‐C bonds. For aromatic SAMs, β_1_ is 0.2–0.4 Å^−1^,^32^, ^33^, ^34^, ^35^, ^36^ and a value of *d*_2_ of 0.9 Å is sufficient to reduce the local current by a factor of 10, and, as mentioned above, changes in the dihedral angle of the two phenyl groups can also induce changes in the current. However, our results show that the SC‐PEP molecules have significantly reduced blinking events than the SC_12_ case in the measured frequency range. Therefore, we conclude that changes in the dihedral angle fall out of the measured frequency window.^63^, ^64^ We note that the dihedral angle can be chemically fixed to remove this degree of freedom and obtain stable conduction through the molecule.^61^

We note that others have performed noise spectroscopy of current time traces of single molecule junctions.^23^, ^25^, ^26^, ^29^, ^70^, ^71^, ^72^, ^73^ In those experiments, the *S*(*f*) analysis was performed in the frequency range of 0.1–10^6^ Hz, and the fluctuation of the tunneling current was attributed to changes in the molecule–electrode (Au‐S) bond,^25^ charge trapping at localized states (or defects),^70^ or the change of molecule–electrode coupling through metal atom movements.^26^, ^73^ In contrast, we performed *S*(*f*) analysis of the light emission from large‐area STJs (in the frequency range of 10^−3^ to 1 Hz) which provides a new way to visualize and examine the local current fluctuations within a large‐area STJ, which is not possible with noise spectroscopy based on the tunnel current measured across the whole junction.

In our experiments, leakage radiation microscopy allows us to directly visualize the effective electrical contact of the STJs. This method, however, is diffraction limited and therefore, in principle, we cannot be certain whether a spot represents conduction via a single or a bundle of molecules. To deepen the understanding regarding the effective contact area, we evaluate the conductance of a single emitting spot. In the low bias region (*V* < 0.3 V), the *J*(*V*) curves are linear from which we determined a conductance of the SC_12_ and SC‐PEP junctions of 1.2 × 10^−7^ *G*_0_ and 8.2 × 10^−7^ *G*_0_, respectively (*G*_0_ is the quantum conductance of 7.75 × 10^−5^ S). Considering the number of emitting spots (the spot number at −2.0 V), that is 42 ± 5 for SC_12_ and 8 ± 3 for SC‐PEP junctions (the error bars represent the average determined from >100 measurements; see the Supporting Information), the conductance of each emitting spot is 2.9 × 10^−9^ and 1.0 × 10^−7^ *G*_0_, respectively. These conductance values are similar (within one order of magnitude) to previously reported values obtained from junctions (10^−9^–10^−8^ *G*_0_ for the SC_12_ molecule^33^, ^74^, ^75^, ^76^, ^77^ and 10^−7^–10^−5^ *G*_0_ for the SC‐PEP molecule without the CH_2_ unit between the sulfur and the phenyl unit^76^, ^78^, ^79^, ^80^, ^81^, ^82^) where the SAMs were contacted with a conducting atomic force microscope tip; in these junctions, the SAMs are chemisorbed on the bottom electrode and form a physisorbed contact with the top electrode. Thus, it seems that each spot represents conduction via a single, or very few, molecule(s), a finding that confirms our earlier suggestions.^20^, ^28^

Our results also reveal that the top electrode forms a 5.2 times more efficient contact with the SC_12_ than with the SC‐PEP SAMs in terms of effective contact area (i.e., the number of spots). We believe that this difference can be explained by the different stiffness of the molecules as molecules with a large conformational degree of freedom can form a better conformal contact with the top electrode than stiff molecules. For techniques that require spin coating of top‐electrodes or protective barriers (that are placed between the SAM and the top electrode), such as organic semiconductors^83^ or graphene (oxide) inks^84^ from solution, differences in surface tension may also play a significant role in SAM//electrode interface formation.

## 3 Conclusions

In conclusion, we demonstrate control over the intermittent light emission of STJ‐based plasmon sources by changing the flexibility of the molecular backbones. Stable light emission (originating from radiative decay of surface plasmons) is obtained for junctions with stiff conjugated molecules, in contrast to the same junctions but with aliphatic molecules. We found that light emission from STJs with aromatic molecules follows a 1/*f* behavior, while STJs with aliphatic molecules show an extra 1/*f* ^2^ component representing the blinking induced by the conformational changes of the molecules. In addition, our methods allow for direct visualization and quantification of the effective electrical contact area making quantitative comparisons to single molecule experiments possible and dynamic characteristics of the molecule–electrode interfaces of molecular tunnel junctions in operando. Thus, we disentangled two sources of heterogeneity—spatial and temporal—and showed that rigidity of the molecules—an inherent property of all molecules—within the junction directly influences the light emission during electron tunneling at the electrical contact areas.

We believe that our results are important for future studies where, for instance, strong intermolecular interactions could be explored to reduce blinking, or how molecular dipoles or charging of the SAMs via redox reactions could affect the molecule–electrode interactions and consequently the blinking dynamics.

## Conflict of Interest

The authors declare no conflict of interest.

## Supporting information

Supplementary

Supplementary

Supplementary
