# Supplementary material for: In Operando Characterization and Control over Intermittent Light Emission from Molecular Tunnel Junctions via Molecular Backbone Rigidity
Source: Adv Sci (Weinh). 2019 Aug 22;6(20):1900390. doi: 10.1002/advs.201900390 (PMC6794720; doi:10.1002/advs.201900390)
Supplement: Supplementary file 1 — Supplementary [file ADVS-6-1900390-s002.pdf]

Copyright WILEY-VCH Verlag GmbH & Co. KGaA, 69469 Weinheim, Germany, 2019.

Supporting Information

for *Adv. Sci.,* DOI: 10.1002/advs.201900390

In Operando Characterization and Control over Intermittent Light Emission from Molecular Tunnel Junctions via Molecular Backbone Rigidity

*Tao Wang, Wei Du, Nikodem Tomczak, Lejia Wang, and Christian A. Nijhuis**

Supporting Information for

In Operando Characterization and Control over Intermittent Light Emission from Molecular Tunnel Junctions via Molecular Backbone Rigidity

Tao Wang[1,5], Wei Du[1], Nikodem Tomczak,[1,2] Lejia Wang[1] and Christian A. Nijhuis[1,3,4]*

[1]Department of Chemistry, National University of Singapore, 3 Science Drive 3, 117543, Singapore, Singapore

[2]Institute of Materials Research and Engineering, A*STAR (Agency for Science, Technology and Research), 2 Fusionopolis Way, Innovis, 138634, Singapore

[3]Centre for Advanced 2D Materials and Graphene Research Centre, National University of Singapore, 6 Science Drive 2, 117546, Singapore, Singapore

[4] NUSNNI Nanocore, National University of Singapore, 117411, Singapore, Singapore

[5]Present address: Institute of Functional Nano & Soft Materials (FUNSOM), Jiangsu Key Laboratory for Carbon-Based Functional Materials & Devices, Soochow University, Suzhou 215123, Jiangsu, China.

Sample preparation

We followed previously reported procedures to prepare the SAM-based tunnel junctions (STJs)[S1-S3], including the preparation of the template-stripped gold substrate (Au^{TS}) as the bottom electrodes, the two types of SAMs, and the EGaIn top electrode stabilized in a PDMS through-hole. For the Au^{TS} substrates, clean coverslip glass slides (Paul Marienfeld GmbH, 22 × 22 mm^2, 0.16–0.19 mm thick) were glued to gold films (50 nm) deposited on silicon wafers with an optical adhesive (OA, Norland, no. 61) to form the glass–OA–Au composite. The OA was cured with ultraviolet light (100 W) at a distance of 40 cm from the substrates for 1 hour. After curing, the glass–OA–Au composites were cleaved off the Si wafer to yield the Au^{TS} substrates. To form the SC_{12} SAMs, we prepared a 3 mM ethanolic SC_{12} solution and purged it with N_2 for 15 mins to remove most of the O_2. To minimize the surface contamination, the Au^{TS} substrates were immersed in the ethanolic SC_{12} solution within 5 s, and the SAMs were formed over a period of time of 3–6 h at room temperature. The SC-PEP SAMs were prepared in a similar way as the SC_{12} SAMs, but with a formation time of 18 h. For the EGaIn top electrode, we used the PDMS microfluidic device to constrain the EGaIn following a previously reported procedure.[S2] The Ga_2O_3/EGaIn top electrode was stabilized in a through-hole that defined the geometrical contact area of the tunnel junction (1000 μm^2). Finally, STJs were formed by simply placing the top electrode on the SAM-modified Au^{TS} substrates.

Electro-optical measurements

We followed light emission from the two kinds of STJs in real-time using an inverted optical microscope (Nikon Eclipse Ti-E) equipped with an electron-multiplying charge coupled device (EMCCD, iXon Ultra 897) and an oil immersion objective (100× magnification, numerical aperture NA = 1.49). The optical setup has been described in detail

elsewhere.[S1] The electrical measurements were performed using the Keithley 6430 source meter with a home-made Labview program to scan the applied voltage and record the J(V) curves or apply the constant voltage and record the corresponding current as a function of time. Simultaneously with the applied bias, we recorded the videos of the light emission from the STJs with the 300 EM Gain using the EMCCD. The integration time for one frame is 1 s for the images in Fig. 3 and 0.5 s for the case in Figs. 4a-b and Fig. 5a.

Rigidity of molecular backbones

We define the "rigidity" of the molecule as the difficulty to bend the molecular backbone. The SC_{12} molecules are in the lowest energy state in the all trans conformation (Fig. 1a). The energy for rotation around the C-C bond is low. For butane, the energy barrier for rotation around the C-C bond is 16-19 kJ/mol (2.5-3.0 × 10^{-20} J per molecule).[S4] Therefore, inelastic tunneling events can induce conformational changes of the molecules involving C-C bond rotations and Gauche defects as explained in the main text. In SC-PEP molecules, the phenyl groups can rotate along the molecular backbone axis, such a rotation, however, will not bend the molecular backbone or disconnect the molecule from the top electrode. It should be noted that the "CH_2" group introduces some flexibility to the SC-PEP molecules. However, we used the SC-PEP precursors with the "CH_2" group because they form SAMs which higher packing density than the precursor without the "CH_2" group. We have studied the packing structure of both types of SAMs in detail in ref. S5 with electrochemistry, X-ray photoelectron spectroscopy (XPS), ultra-violet photoemission spectroscopy (UPS) and near edge X-ray adsorption fine structure spectroscopy (NEXAFS); this study clearly shows that the SAMs without the CH_2 unit are inferior and suffer from disordered domains with flat lying molecules while SAMs derived from SC-PEP form dense structures with all the molecules in the standing up phase.

Reproducibility of the experiments.

Our conclusions are based on 8 different junctions made for each type of SAM (16 junctions in total) and 5-10 measurements per junction (more than 100 measurements in total). Figures S1 and S2 show the light emission images observed from all 8 SC_{12} and 8 PEP junctions recorded at -2.0 V. Here, the number of light emission spots of the SC_{12} junctions is 42 ± 5, in contrast to the number of light emission spots of 8 ± 3 for the SC-PEP junctions where the error indicates the standard deviation 120 and 110 measurements, respectively.

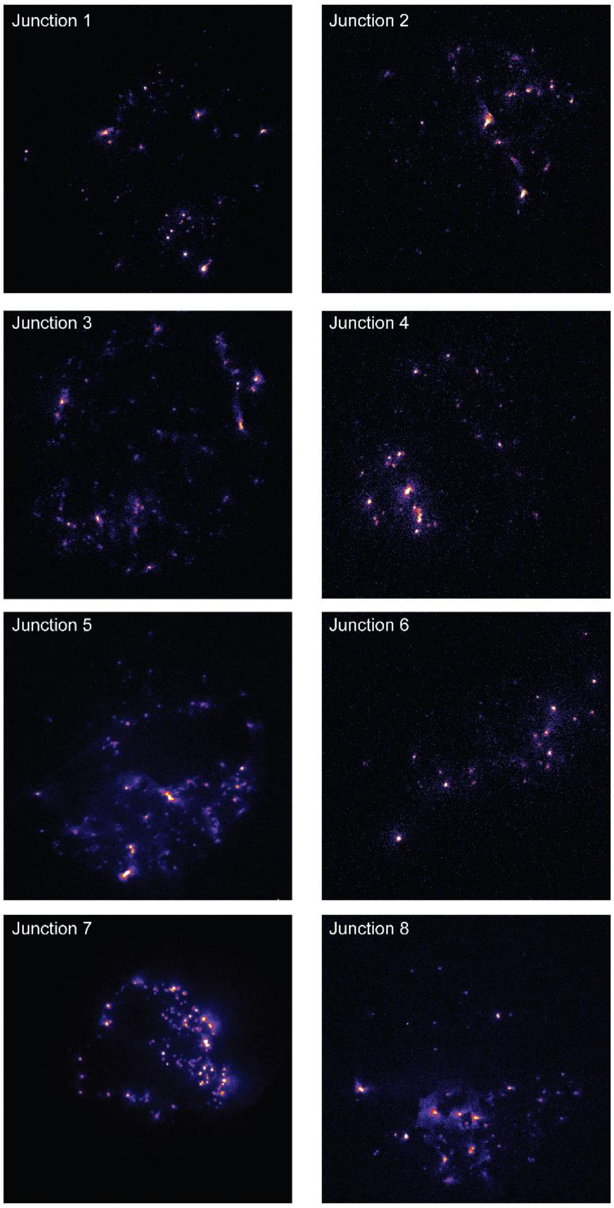

Figure S1. Light emission images from 8 STJs with the SC_{12} SAM.

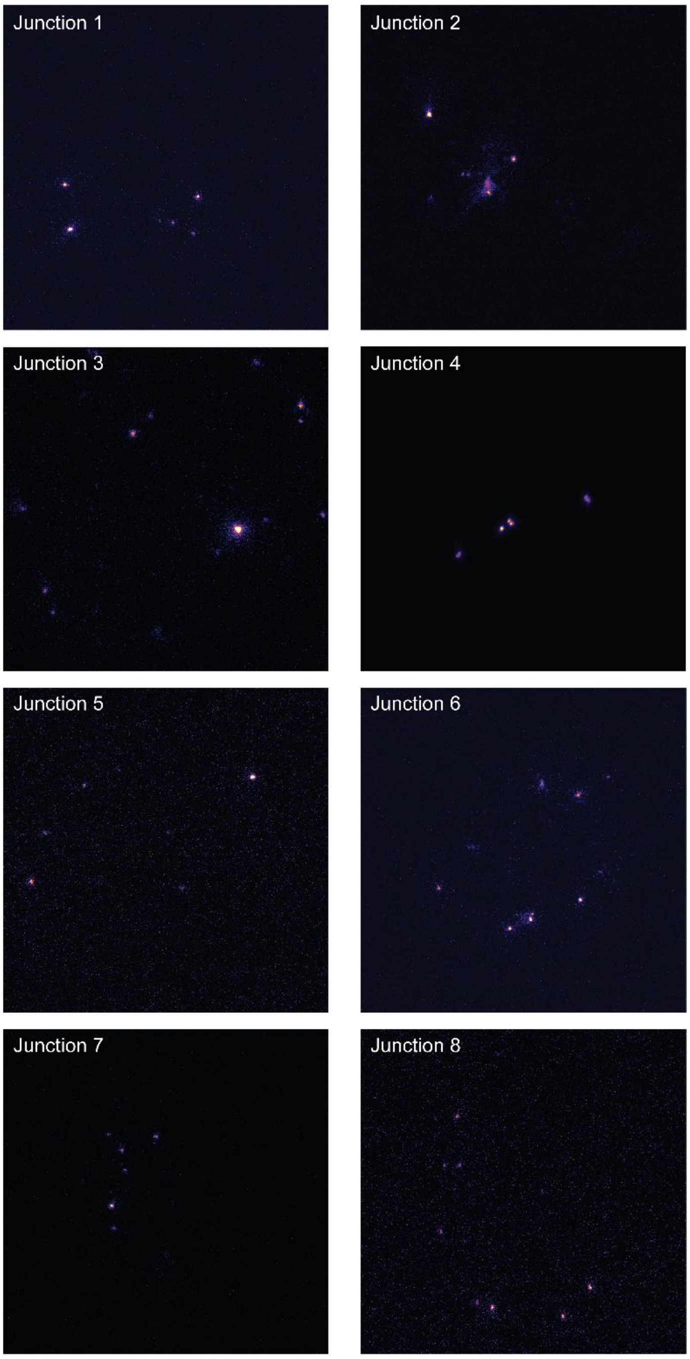

Figure S2. Light emission images from 8 STJs with the SC-PEP SAM.

Spectra of light emission of the SC-PEP junction

Figure S3 shows the light emission spectra from SC-PEP SAMs. The spectrum is voltage dependent and is similar to the light emission spectra observed from SC_{12} SAMs.[S1]

It should be note that although the bandgap of the SC-PEP molecules is lower than that of the SC_{12} molecules, the HOMO-LUMO gap is still about 4.5 eV (Table 1 in ref. S6). In our optical characterization experiments, we used a bias up to 2.2 V which is not large enough for electroluminescence. We also note that GaOx has a bandgap of 5 eV (ref. S7), far too large to result in photon emission in the bias window of up to 2 V we used.

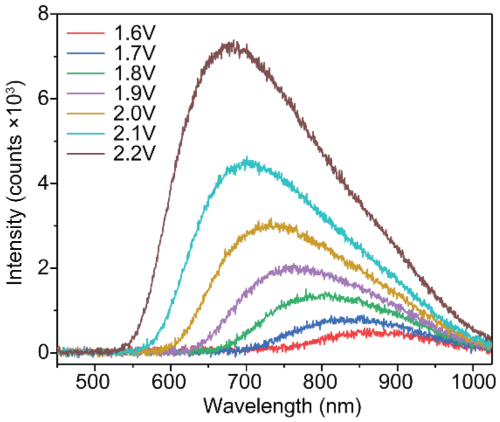

Figure S3. Light emission spectra of the SC-PEP junction.

Bias dependent *S*(*f*) of the light emission from SC-PEP junctions

Similar to Figure 5 in the main text, we also investigated the bias dependent *S*(*f*) of the light emission from the SC-PEP junctions. Figure S4 shows that the blinking events for SC-PEP junctions also slightly increase when the applied bias increases from -1.8 V to -2.2 V. Relative to SC_{12} STJs, the blinking events in the intensity time traces are more than 100 times lower and *S*(*f*) (Fig. S4b) is dominated only by the 1/*f* component.

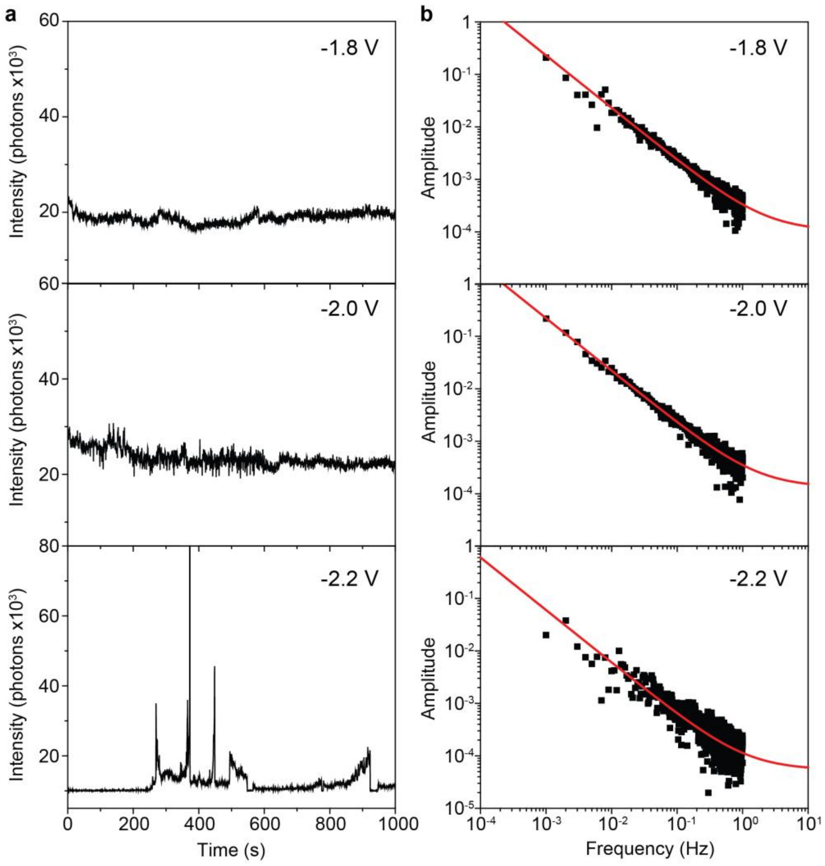

Figure S4. (a) Emission intensity time traces of the SC-PEP junction recorded at -1.8 V, -2.0 V and -2.2 V. (b) The corresponding *S*(*f*) analysis of the emission intensity time traces in a.

Details of the *S*(*f*) analysis

We collected the time trace of the light emission intensity ranging from 0.5 to 1000 s with an interval of 0.5 s (step 1, Fig. S5). Next, we calculated the autocorrelation of these time traces to determine the lag time ranges from 0.5 s to 999.5 s at 0.5 s intervals (step 2, Fig. S5). No filter was applied and no normalization was used. Finally, the FFT of the autocorrelation was obtained, also here no filter nor normalization was used. The amplitude

of the FFT represents the power spectral density *S*(*f*) which was fitted to eq.1 (step 3, Fig. S5).

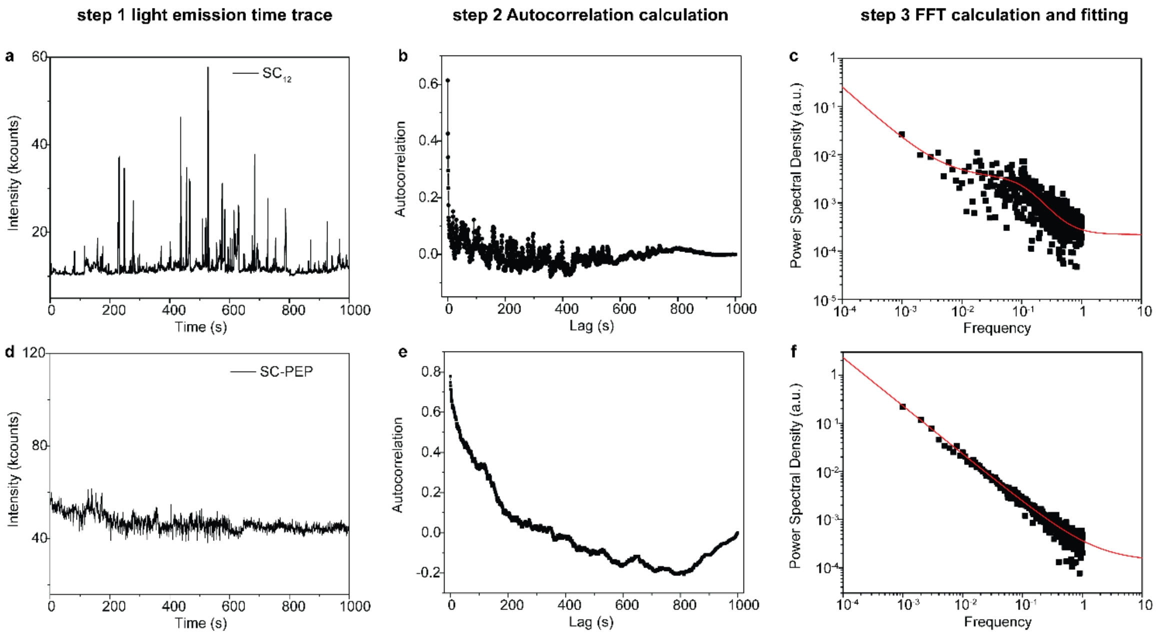

Figure S5. Work flow of the *S*(*f*) analysis.

Time traces of the current and photon emission intensity

Figure S6 shows the time traces of the total current and the total intensity from the entire junction area of 4 SC_{12} junctions. There are variations in the time traces for the total current and the total intensity, but these variations are smaller than a factor of 2. These small variations are dramatically different from the blinking of single emission spots, where the light emission intensity changes 5-10 times. From these traces molecular events cannot be determined.

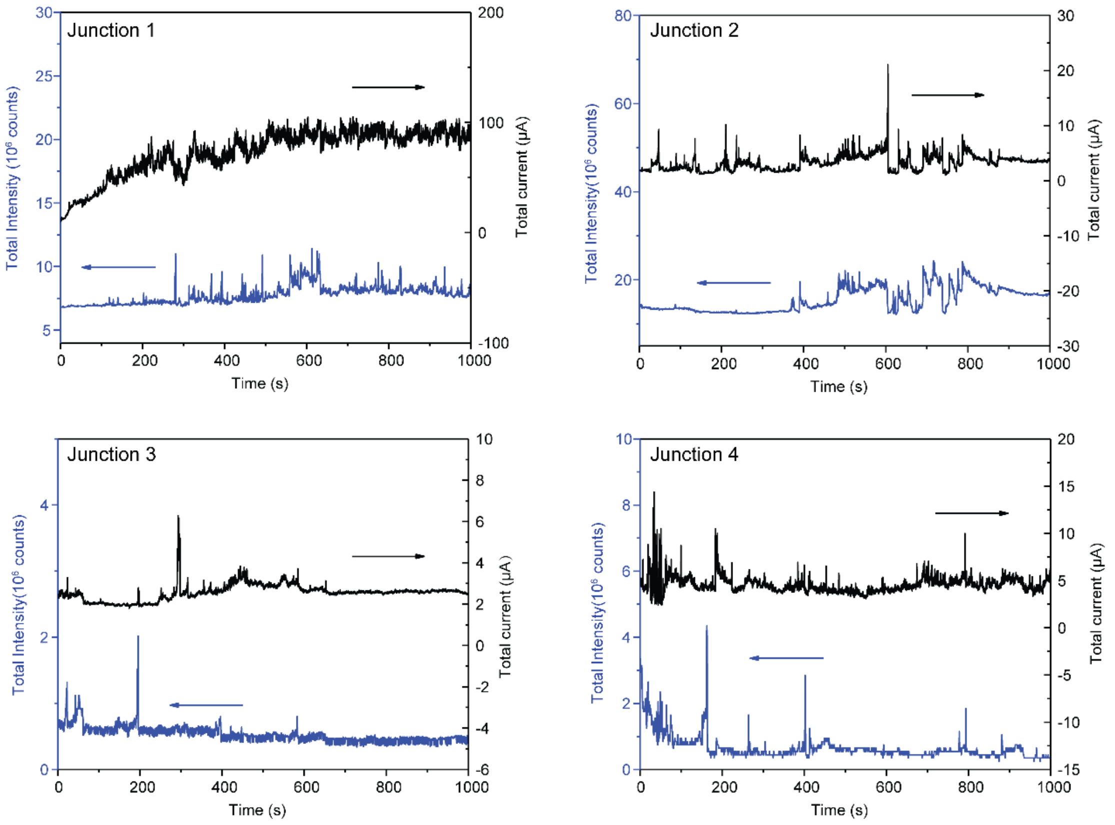

Figure S6. Time traces of total current and total photon emission intensity of 4 SC_{12} junctions at -2.0 V.

References

[S1] Du, W.; Wang, T.; Chu, H. S.; Wu, L.; Liu, R.; Sun, S.; Phua, W. K.; Wang, L.; Tomczak, N.; Nijhuis. C. A. On-chip molecular electronic plasmon sources based on self-assembled monolayer tunnel junctions. *Nature Photon*. **2016**, 10, 274–280.

[S2] Wan, A.; Jiang, L.; Sangeeth, C. S. S.; Nijhuis, C. A. Reversible soft top-contacts to yield molecular junctions with precise and reproducible electrical characteristics. *Adv. Funct. Mater.* **2014**, 24, 4442–4456.

[S3] Jiang, L.; Wang, T.; Nijhuis, C. A. Fabrication of ultra-flat silver surfaces with sub micro-meter scale grains. *Thin Solid Films* **2015**, 593, 26–39.

[S4] Smith, J. G. Organic Chemistry, 4th ed.; McGraw-Hill Education, 2013.

[S5] L. Wang, L. Yuan, L. Jiang, X. Yu, L. Cao, C. A. Nijhuis. *J. Phys. Chem. C* **2019**, DOI: 10.1021/acs.jpcc.9b05614

[S6] Z. Tang, R. K. Hicks, R. J. Magyar, S. Tretiak, Y. Gao, H. L. Wang, *Langmuir* **2006**, 22, 8813–8820.

[S7] W. Zhang, B. S. Naidu, J. Z. Ou, A. P. O'Mullane, A. F. Chrimes, B. J. Carey, Y. Wang, S.Y. Tang, V. Sivan, A. Mitchell, S. K. Bhargava, K. Kalantar–zadeh. *ACS Applied Materials & Interfaces* **2015**, 7, 1943–1948.
